# Does ‘Fear of COVID-19’ trigger future career anxiety? An empirical investigation considering depression from COVID-19 as a mediator

**DOI:** 10.1177/0020764020935488

**Published:** 2020-07-02

**Authors:** Md. Shahed Mahmud, Mesbah Uddin Talukder, Sk. Mahrufur Rahman

**Affiliations:** 1 Department of Management, Mawlana Bhashani Science and Technology University, Tangail, Bangladesh; 2 Department of Food Technology and Nutritional Science, Mawlana Bhashani Science and Technology University, Tangail, Bangladesh; 3 Department of Business Administration, North Western University, Khulna, Bangladesh

**Keywords:** Fear of COVID-19, career anxiety, depression from COVID-19, structural equation modeling

## Abstract

**Background:**

Due to the outbreak of COVID-19, the mental health of the people all around the world is severely disrupted.

**Aim:**

The purpose of this study is to identify whether ‘Fear of COVID-19’ impacted on future workforces’ career anxiety at the first place and whether depression from COVID-19 has any indirect effect on ‘Fear of COVID-19’ and future workforces’ career anxiety.

**Method:**

Based on three different scales related to ‘Fear of COVID-19’, depression and career anxiety, a structured questionnaire was developed and the survey data was collected for this study.

**Results:**

The empirical result of the study reveals that, due to the outbreak of COVID-19 fear, the future workforce is getting anxious about their future career. Again, depression from COVID-19, caused by ‘Fear of COVID-19’, as a mediator, has a significant indirect impact on the relationship between ‘Fear of COVID-19’ and future career anxiety, resulting in a full mediation. This means, due to the outbreak of ‘Fear of COVID-19’ people are becoming depressed and anxious about their future career which is creating a long-term negative effect on human psychology.

**Conclusion:**

These research findings will be a major tool for the policymakers, as well as the human resource planning professionals, to sketch plans after COVID-19 pandemic. This study is a novel work combining the concepts of fear and depression with career anxiety in a pandemic situation like COVID-19, and also assists future researchers in many folds.

## Introduction

‘Pandemics are large-scale epidemics afflicting millions of people across multiple countries, sometimes spreading throughout the globe’ (World Health Organization (WHO), 2010). Over the last couple of centuries, a number of pandemics have been reported causing threats for humankind (Taylor, 2019). On 11 March 2020, the WHO officially declared COVID-19 as a universal pandemic (WHO, 2020d). Prior to the declaration, China officially reported the virus in late 2019 (Moghanibashi-Mansourieh, 2020). WHO labeled this novel coronavirus as the sixth public health emergency of international concern (Moghanibashi-Mansourieh, 2020). As of 28 May 2020, the mortality rate was 6.32% worldwide, where a total number of confirmed and death cases were 5,593,631 and 353,334, respectively, and a total of 216 countries, areas or territories had confirmed cases (WHO, 2020a). Clinical research suggests that fever, dry cough and tiredness are some of the common symptoms, whereas aches and pains, nasal congestion, diarrhea, smell and taste disorders, breathing problem and so on are some of the exceptional symptoms of novel coronavirus (Guan et al., 2020; Holshue et al., 2020; WHO, 2020c). Pandemics like coronavirus create not only an epidemiological crisis but also a psychological crisis (i.e., anxiety, depression, insomnia, trauma, anger, psychosis, panic and boredom) like the other pandemics in the past (American Psychological Association, 2020b; Balaratnasingam & Janca, 2006; Brooks et al., 2020; Özdin & Bayrak Özdin, 2020; Tucci et al., 2017; WHO, 2020b). A fresh poll conducted by Kaiser Family Foundation (KFF; 2020) claimed that nearly half of the respondents are facing some kind of mental health–related issues in this novel corona virus pandemic, which gave a similar kind of results from the past (Kanadiya & Sallar, 2011; Rubin et al., 2009).

With the spread of COVID-19, governments are taking crude measures to halt the spread of the virus. This new global pandemic has halted public life and is grinding the global economy hard (Bakker & Wagner, 2020). Industries and economies in the universe are facing an unprecedented challenge to survive. A wide number of industries already laid off their employees and some others are thriving (Pappas, 2020). Hundreds of thousands of people already lost their jobs by being dismissed and some others are feeling the heat of COVID-19 hard (Charles & Anderson-Nathe, 2020; Coibion et al., 2020; Lee et al., 2020; Sarkar, 2020). The global demand for products has plummeted, and the world economy is on the brink of great financial depression (Duffin, 2020; Goodman, 2020; Menickella, 2020; Oxford Economics, 2020; Riley, 2020; Sheppard et al., 2020). In a recent report, the International Monetary Fund (IMF) predicted that the upcoming economic depression will break the records of 1930s Great Depression and ‘many countries face a multi-layered crisis comprising a health shock, domestic economic disruptions, plummeting external demand, capital-flow reversals and a collapse in commodity prices’ due to the outbreak of COVID-19 (*BBC*, 2020; Martin & BLOOMBERG, 2020; Perez, 2020; Wiseman & Crutsinger, 2020). Thus, in this uncertain situation, the fear of economic and social anxiety popped-up into the mind of people resulting in lots of psychological and physiological illnesses in parallel (Yetgin & Benligiray, 2019). Social media and some other new forms of non-traditional media somehow worsen the crisis. Lack of control over rumors and misleading and untrusted information fuels the fear of COVID-19 in a much wider range, which was not seen before (Bora et al., 2018; Moghanibashi-Mansourieh, 2020; Sharma et al., 2017; Walker, 2016; Weir, 2020). As a result, people are getting anxious, depressed, frustrated and stigmatized, and this uncertain-like situation leads many of them toward suicide (Goyal et al., 2020; Lai et al., 2020; Lee, 2020; Lee et al., 2020; Mamun & Griffiths, 2020; Pappas, 2020; Xiang et al., 2020). Taylor (2019) argued that during the outbreak of infectious diseases some clinical signs of fear and anxiety were found. Again, there exists a very close connection among fear, anxiety and depression (Taylor, 2019). Anxiety is a state of mental feeling of tension and worries about the future (Banerjee, 2020). Because of COVID-19, the economy is shrinking worldwide, thus becoming a major concern for the current and future workforce. Fear about getting jobs and sustaining existing jobs somehow form career-related anxiety into the mind of current and future workforce. Financial analysts are warning about the upcoming recession caused by COVID-19, which is creating a new form of a mental burden for the current and potential workforce to think about their future (Lee et al., 2020). On this backdrop, this research led by the following objectives:

a. to examine the impact of ‘Fear of COVID-19’ on future workforces’ career anxiety and
b. to find the mediating role of depression from COVID-19 in the relationship between ‘Fear of COVID-19’ and future workforces’ career anxiety.

Like any other pandemic situation, people tend to fear their life in the first place and then the fear of their belongings also arises. As the fear deepens, that ultimately forms depression and both fear and depression relate to different forms of anxiety. Based on the assumption, this research tries to understand the direct and indirect relationship between ‘Fear of COVID-19’ and career anxiety where depression from COVID-19 plays the role of a mediator. By adopting and applying three different scales on ‘Fear of COVID-19’, depression and career anxiety, this study examines a structural relationship to test the assumptions which is unique until now. The rest of the article is structured as follows. First the literature review and hypothesis development is presented. Next, the methodology, data analysis and discussion are offered. Finally, the article ends with limitations, conclusion and implications.

## Literature review and hypothesis development

### Theories of emotion

While forming human behavior, emotion has a profound impact (Cherry, 2019). Cherry (2019) described emotion as ‘a complex state of feeling that results in physical and psychological changes that influence thought and behavior’. Emotion is a complex set of processes that evolve with the passage of time as an evolutionary theory of emotion suggests (Cherry, 2019). Different researchers, psychologists and philosophers describe different forms of emotional aspects and theories, which plays a significant role to understand the behavioral outcome of human. Among them, the James-Lange theory of emotion, Cannon-Bard theory of emotion, Schachter-Singer theory of emotion and Lazarus theory of emotion are some of the dominant theories of emotion. The James-Lange theory postulates that persons’ emotional response is influenced by the physiological response, which means there shall be an event and that event impacted persons’ physiology and thus the emotion will work for that person (Walsh, 2013). The Cannon-Bard’s theory raises some questions over the James-Lange theory and argued that, without building up emotion, the physiological response cannot be possible. So, this theory hypothesized that there shall be an event at the first place and then both the physiological response and the experience of emotion occurred simultaneously (Cherry, 2019; Walsh, 2013). Again, the Schachter-Singer’s theory of emotion assumes that, after facing an event, the physiological stimulation happens first, then individuals have to identify the reason behind stimulation to experience and marked it as an emotion (Cherry, 2019; Walsh, 2013). Finally, according to the Lazarus’s theory of emotion, there shall be an event and the person first labels (appraise) the event, and this leveling is basically done through a person’s personal experiences or shaped by culture, religion or other factors. Based on the labeling, one’s emotion will be formed and physiological response occurs almost instantaneously (Walsh, 2013). Thus, all forms of emotional state (i.e., fear, anger, anxiety, depression, trauma, psychosis, panic, boredom, etc.) of humans can be linked with these prominent theories of emotion. The current study extends the theories of emotion by examining the impact of ‘Fear of COVID-19’ on future career anxiety and the mediating role of depression from COVID-19 in this relationship.

### ‘Fear of COVID-19’ and future career anxiety

Human emotion is a complex set of processes, where fear and anxiety play a great role independently. Based on phenomenology, behavioral expressiveness, psychometric and the level of neurobiology, the human emotion of fear and anxiety are essentially different from each other (Barlow, 2000, 2002; Barlow et al., 1996; Bouton et al., 2001). From a theoretical viewpoint, fear has been considered as a primary form of emotion that is present commonly across ages, races, cultures and species. Beck and Emery (1979) defined fear as ‘awareness and appraisal of danger, and anxiety as the unpleasant feeling state and physiological reaction that occurs when fear is provoked’. A new and fatal epidemic disease like COVID-19 can form fear, panic, anxiety and stigma very quickly among individuals worldwide (Ahorsu et al., 2020; Lee et al., 2018; Strong, 1990). With access to real-time information, unauthentic pieces of information and upsurge of social media rumors somehow create a new breed of fear in this pandemic situation (Abramson, 2020; Lin, 2020; Rubin & Wessely, 2020). With the presence of this fear and the uncertainty about the future, the human emotional system forms some kind of anxiety. Anxiety is a state of human emotion which can be formed due to the perception of future threat (Dobson, 1985). Epstein (1985) portraits anxiety as ‘threats to future happiness, threats to self-esteem, and threats to the individual’s ability to make sense of the data of his experience’. Izard (1977) identified anxiety as a fusion of different emotional statuses of the human mind, although fear remains a distinct part of that fusion. Izard (1977) further argued that the blend of anxiety may be changed with the change of status quo and time. Uncertainty about the future career often impacted an individual’s present quality of life, resulting in some kind of anxiety (Mostert & Botha, 2013). During the time of the global pandemic, career-related anxiety gets momentum particularly among the forthcoming university graduates who are going to face the job market in the near future. According to the theory of career development, it is established that university students ages 23 to 25 years are forming their career expectations and career commitments (Super, 1980; Tsai et al., 2017). But due to the outbreak of any unwanted global phenomenon the regular expectation level comes down and the situations like COVID-19 fear poses the greatest threat for those minds who are planning their future career (Tsai et al., 2017). Thus, in this uncertain situation, the potential workforces’ fear turned toward career anxiety and they cannot make any fruitful decision about the future (Hornak & Gillingham, 1980; Kaplan & Brown, 1987). Hence, the researchers hypothesize as follows:

*H_1_: There exists a significant relationship between ‘Fear of COVID-19’ and future workforces’ career anxiety*.

### The mediating role of depression from COVID-19

**Figure 1.**
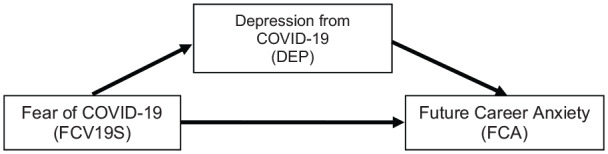
Proposed research model.

Fear, depression and anxiety, all are playing a significant role in the study of emotion. Barlow (2000) identified fear as the alarm system of human psychology, whereas most of the prominent philosophers and psychologists believe that the spectacle of anxiety and depression are deep-rooted on the fundamental concepts of human emotion (Barlow, 2002; Izard, 1971, 1977; Klerman, 1977; Plutchik, 1980). Normally, fear occurs when people are directly threatened with a dangerous event (i.e., life-threatening), and anxiety is ‘a state of helplessness, because of a perceived inability to predict, control, or obtain desired results or outcomes in certain upcoming personally salient situations or contexts’ (Barlow, 2000). However, depression is a psychological disorder which comprises a persistent feeling of sorrow and loss of interest (American Psychological Association, 2020a; *Medical News Today*, 2020b). Some of the research studies conducted by Dobson (1985), Spielberger et al. (1970), Zung (1965) and others found that there exists a very close relationship between anxiety and depression. Although some forms of differences exist by definition among the fear, anxiety and depression, different emotional and cognitive models, as well as psychometric-based literature, announce the empirical relationship among fear, anxiety and depression (Dobson, 1985; Izard, 1977). Izard (1977) found a close connection among fear, anxiety and depression and further pointed out that fear is mostly concentrated with future emotions, whereas anxiety predicts the threat of future and depression related to the responses of past or imminent events (Dobson, 1985). Due to the outbreak of COVID-19, the fear arises among the future workforce regarding their future career plan, which ultimately triggers career-related anxiety among them. An increasing number of job loss and the uncertain future job market grow depression among the future workforces’ mind as the virus may last longer and dries up the world’s economy more stronger than expected (*Medical News Today*, 2020a; Morath & Guilford, 2020; *UN News*, 2020; *The Wall Street Journal*, 2020). Thus this research assumes that due to the COVID-19 pandemic, fear grows among the future workforce, ‘Fear of COVID-19’ has a direct influence on building career-related anxiety among the future workforces’ mind and depression from COVID-19 has a mediating role over that direct relationship (Figure 1) as the unknown long-term effect of COVID-19 fear threatening the future. Hence, this research hypothesized the following:

*H_2_: Depression from COVID-19 mediates the relationship between ‘Fear of COVID-19’ and future workforces’ career anxiety.*

## Methodology

The population for this study is the current university-going regular students from different public universities of Bangladesh who are unemployed right now and will be facing the job market within the next 6 months to 12 months. That is why this study employed a purposive sampling technique (non-probability sampling) for sample selection. To meet the objective and population dimension of this research, students studying only in the fourth year (final semester) or master’s level at different public universities of Bangladesh are taken into consideration for the sample. For performing a structural equation modeling (SEM) analysis at least 150 sample size are required for this study, as the conditions suggested by Hair et al. (2019, p. 633). Through a structured self-administrated online questionnaire (Google form) data was collected from the sample. Initially, the questionnaire was developed in English in light of the scales adopted for this study. Then the English version questionnaire was translated into Bangla language for data collection purposes (the Bangla version questionnaire for depression scale adopted from Alim (2018)), as Bangla is the mother tongue of the target population. In the process of translating tasks, language experts were involved in maintaining the translation quality and the validity of the questionnaire. Finally, an online-based survey was conducted among the targeted population. The questionnaire link, with required instructions, was disseminated in different Facebook messenger groups of the targeted population. The data was collected in the last week of April 2020. A total of 246 responses were collected during the time period. From 246 responses, 232 (94.31%) responses met the criteria and 14 responses was dropped due to straight-lining (Kim et al., 2019) and missing information. The questionnaire had two parts. Part A collected the demographic-related information, and Part B collected the response of the items of three latent variables used in this study. All the measures used in this study contained items with five-point Likert-type scales ranging from 1 = *strongly agree* to 5 = *strongly disagree*. For this study purpose, Helsinki’s declaration of research ethics was followed. Besides, the study was approved by the Department of Business Administration of Mawlana Bhashani Science and Technology University’s ethics committee. The online survey questionnaire contained the purpose and outcome of the study and also the rights of the respondents. All participants provided informed consent where the anonymity and confidentiality of the data were maintained strictly. IBM SPSS software was used for importing the data from the online survey and for preliminary analysis while multivariate analysis was done using IBM AMOS 24 software.

## Measurements of variables

### Fear of COVID-19 Scale (FCV19 S)

On the backdrops of COVID-19 outbreak, Ahorsu et al. (2020) developed and validated a scale called the ‘Fear of COVID-19 Scale’ (FCV-19 S) – a seven-item scale especially developed for measuring the fear of novel coronavirus (see Appendix 1) – and used as an independent variable in this study.

### Future career anxiety (FCA)

Due to the outbreak of any uncertain situation like COVID-19, people tend to get anxious about their future, and this anxiety escalates much more if there is a fear of death and future livelihood. Pandemic like COVID-19 broke the hope of the university students who are preparing themselves for the job market in the near future. Thus, prolonged fear turned into anxiety. For this, to measure, future career-related anxiety, this study adopted the measurement items from a previous work related to career anxiety scale developed and validated by Tsai et al. (2017) (see Appendix 1). Here, in this study, this measurement scale has been employed as a dependent variable.

### Depression from COVID-19 (DEP)

Depression is a state of mental position which is a very common phenomenon of human emotion. For measuring depression, a number of scales exist, and different researchers use these scales to measure the state of depression of individuals. Among those scales, DASS scale is one of the widely accepted measurement scales, due to its statistical power, robustness and stability over a longer period of time for measuring depression, anxiety and stress (Brown et al., 1997; Lovibond & Lovibond, 1995; Parkitny & McAuley, 2010; Psychology Foundation of Australia, 2018). Initially, there were 42 items in a scale, but later on, a 21-item scale was developed keeping the original sense unchanged (Psychology Foundation of Australia, 2018). This study adopted a validated six-item depression sub-scale for measuring depression from COVID-19 formed by the ‘Fear of COVID-19’ (see Appendix 1) as a mediator (Shea et al., 2009).

## Empirical analysis

### Demographic profile

Table 1 represents the demographic profiling of the respondents. A total of 232 respondents’ responses were considered for further analysis of this study. From Table 1, it can be seen that 54.7% males and 45.3% females were the respondents for this study. As this research only dealt with the fourth year (undergraduate final semester) and master’s (postgraduate) level students, a maximum number of students lie between the age range of 18 and 25 years. Finally, the undergraduate and postgraduate students’ percentage represent in the table 1.

**Table 1.** Respondent’s descriptive profile (*n* = 232).

Variable	Attribute	Frequency	Percentage (%)
Gender	Male	127	54.7
	Female	105	45.3
Age	18–25 years	220	94.8
	26–40 years	12	5.2
Marital Status	Single	215	92.7
	Married	17	7.3
Current Education Level	Undergraduate (fourth year)	172	74.1
	Postgraduate (Masters)	60	25.9

Source: Output from IBM® SPSS.

### Source of getting information regarding COVID-19

In this research, we tried to explore the relationship among fear, anxiety and depression due to the outbreak of COVID-19. Thus, it is necessary to know the respondents’ source of getting information as the types of media and the validity of information create a different kind of emotional feeling on an individual’s mind.

**Figure 2.**
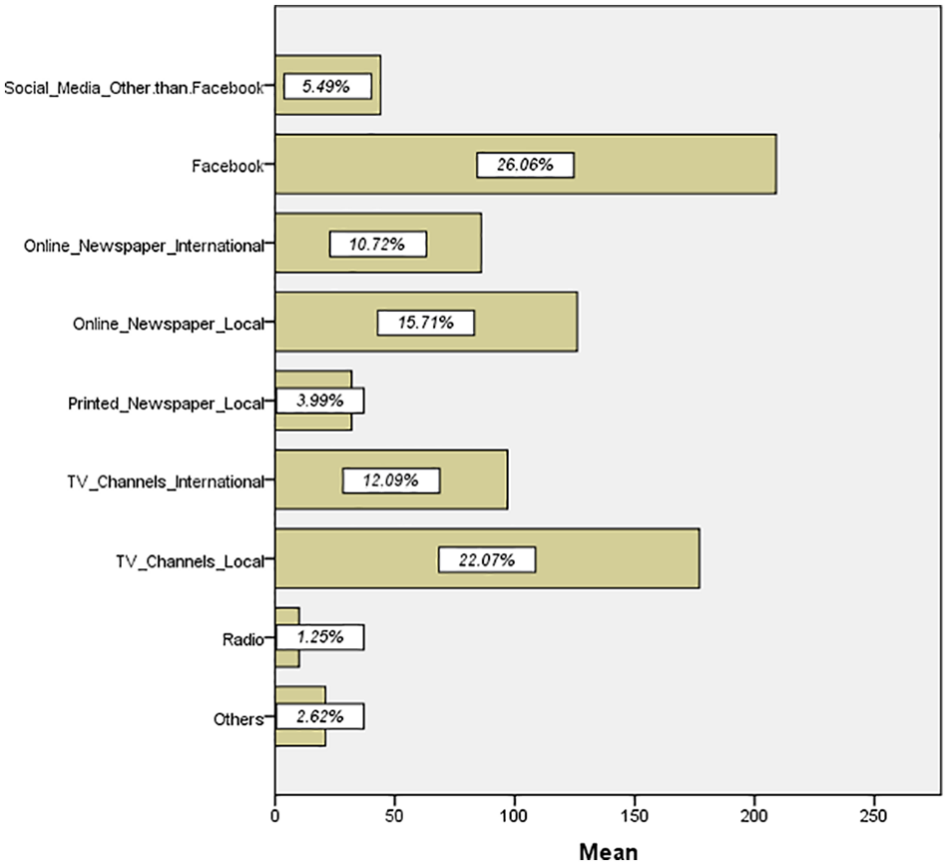
Source of getting information regarding COVID-19. Source: Output from IBM SPSS.

Figure 2 summarizes that most of the respondents prefer Facebook followed by local television channels and newspapers to collect the information about COVID-19.

### Correlation matrix

From Table 2 it can be concluded that there exists a positive relationship among the variables used in this study.

**Table 2.** Correlation Matrix.

Component	DEP	FCA	FCV19 S
DEP	1		
FCA	0.442	1	
FCV19 S	0.576	0.367	1

DEP: depression from COVID-19; FCA: future career anxiety; FCV19S: fear of COVID-19 scale.

Extraction method: principal component analysis.

Rotation method: Promax with Kaiser normalization.

Source: Output from IBM SPSS.

To have a better fit of the model, communalities test was run using IBM SPSS software. From the analysis, one item (FCV19 S4) of ‘Fear of COVID-19’ (FCV19 S) was dropped due to poor communality extraction (0.40).

### The model fit measures

To have a good fit model and to present a structural relationship, it is necessary to measure the relationship between the latent variables and their items. Then the structural relationship can be performed to explore the relationship between latent variables. A total of 18 questions in this study constitute three latent variables. From the 18 questions, 1 item was removed because of poor communality extraction; finally, a total of 17 items/questions are taken into consideration to proceed further. The model fit was tested by different model fit indicators, which is given in table 3.

**Table 3.** Model fit measures.

Measure	Estimate	Threshold	Interpretation
CMIN	230.834	–	–
DF	112	–	–
CMIN/DF	2.061	Between 1 and 3	Within range
CFI	0.94	> 0.95	Acceptable range
SRMR	0.065	< 0.08	Within range
RMSEA	0.068	< 0.06	Acceptable range
PClose	0.011	> 0.05	Acceptable range

CMIN: chi-square value; DF: degrees of freedom; CFI: comparative fit index; SRMR: standardized root mean residual; RMSEA: root mean square error of approximation.

Source: IBM AMOS plugin by Gaskin and Lim (2016).

From Table 3, it can be summarized that this study questions/items of the latent variables pass through all the major model fit indicators suggested by Hair et al. (2019), Malhotra and Dash (2016), Gaskin and Lim (2016) and Hu and Bentler (1999).

### The results of the measurement model

The results for measuring the reliability and validity of the measurement model are illustrated in table 4.

**Table 4.** The items’ estimate and the constructs’ Cronbach’s α, AVEs and CRs.

Constructs	Items	Estimate	Cronbach’s α	Average Variance Extracted (AVE)	Construct Reliability (CR)
Depression from COVID-19 (DEP)	DEP1	0.77	0.88	0.52	0.87
	DEP2	0.72			
	DEP3	0.78			
	DEP4	0.72			
	DEP5	0.68			
	DEP6	0.66			
Future Career Anxiety (FCA)	FCA1	0.82	0.87	0.61	0.88
	FCA2	0.87			
	FCA3	0.85			
	FCA4	0.72			
	FCA5	0.60			
Fear of COVID-19 (FCV19 S)	FCV19 S1	0.53	0.82	0.41	0.80
	FCV19 S2	0.59			
	FCV19 S3	0.66			
	FCV19 S5	0.61			
	FCV19 S6	0.66			
	FCV19 S7	0.74			

DEP: depression from COVID-19; FCA: future career anxiety; FCV19S: fear of COVID-19 scale.

Source: IBM SPSS output, IBM AMOS output and IBM AMOS plugin by Gaskin and Lim (2016).

Table 4 provides various measures of the measurement model. The estimates or standard loading of each item ranges from 0.53 to 0.87. According to Hair et al. (2019) and Malhotra and Dash (2016), the standard loading estimates should be 0.50 or higher (ideally 0.70 or higher). Thus, all the item’s loading passes through the minimum criteria, and some of them are well above the ideal criteria. Again, Cronbach’s α is a reliability measurement criterion which is ranging from 0 to 1, and 0.60 is the lower boundary prescribed by Hair et al. (2019). From the above table, it is seen that all three values of Cronbach’s α is well above the minimum criteria (> 0.80). Finally, for average variance extracted (AVE) and construct reliability (CR), Hair et al. (2019) suggested that the minimum criteria should be 0.50 and 0.70 consecutively although Malhotra and Dash (2016) argued that ‘AVE is a more conservative measure than CR’, so on the basis of the value of CR it can be concluded that the constructs are valid and reliable. Thus, Table 4 represents that the reliability and validity of the constructs applied in this study met the criteria.

### The results of the structural model

Table 5 shows the results of the structural model. From the result, it is found that with a direct effect of ‘Fear of COVID-19’, there is a significant impact established on future workforces’ career anxiety. Thus, H_1_ is statistically supported.

**Table 5.** The result of the structural model.

Hypothesis	Paths			Estimate	S.E.	C.R.	P	Result
Effect of Fear of COVID-19 on Future Career Anxiety (Before Mediation)								
H_1_	FCA	<—	FCV19 S	0.692	0.141	4.894	0.000*	H_1_ supported
Effect of Fear of COVID-19 on Future Career Anxiety (After Mediation)								
H_2_	DEP	<—	FCV19 S	1.368	0.21	6.508	0.000*	H_2_ supported with a full mediation
	FCA	<—	DEP	0.371	0.107	3.478	0.000*	
	FCA	<—	FCV19 S	0.198	0.196	1.011	0.312	

FCA: future career anxiety; DEP: depression from COVID-19; FCV19S: fear of COVID-19 scale.

Significance of estimates: * *p* < .001.

Source: IBM AMOS output.

On the other hand, with the presence of a mediator, depression from COVID-19, it can be seen that the relationship between ‘Fear of COVID-19’ and future workforces’ career anxiety becomes insignificant and creates a full mediation effect into the relationship. The indirect relationship and its properties are shown in Table 6 as suggested by Malhotra and Dash (2016).

**Table 6.** Indirect effect of the model.

Indirect Path	Unstandardized Estimate	Standardized Estimate	Lower	Upper	*p* Value
FCV19 S –> DEP –> FCA	0.508	0.325	0.217	0.908	.007*

DEP: depression from COVID-19; FCA: future career anxiety; FCV19S: fear of COVID-19 scale.

Significance of estimates: * p < .01.

Source: IBM AMOS plugin by Gaskin and Lim (2016).

**Figure 3.**
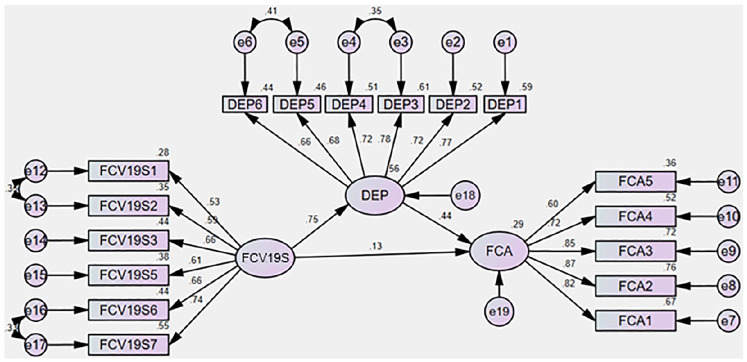
The results of the full model. Source: Output from IBM AMOS.

After performing a bootstrap of 5,000 samples with 95% bias-corrected confidence intervals it is found that with the presence of mediator (depression from COVID-19), the direct relationship between ‘Fear of COVID-19’ and future workforces’ career anxiety becomes insignificant and creates a full mediation relationship. The structural model illustrated in Figure 3.

## Discussion

Due to the spread of COVID-19 pandemic all over the world, a new breed of fear popped up into human emotion especially in the marginal and vulnerable segments of the society. This COVID-19 fear creates a major threat to the future workforce who are planning to serve the job market in the near future. For this, the study hypothesized, ‘Fear of COVID-19’ has a direct impact on future workforces’ career anxiety. Furthermore, depression from COVID-19 mediates the direct relationship between ‘Fear of COVID-19’ and future workforces’ career anxiety. The empirical result reveals that, without the presence of depression from COVID-19, there exists a significant relationship between ‘Fear of COVID-19’ and future workforces’ career anxiety, meaning due to the outbreak of COVID-19, the future workforce are getting anxious regarding their future career. On the other hand, with the appearance of depression from COVID-19, as a mediator, the indirect relationship between ‘Fear of COVID-19’ and future workforces’ career anxiety becomes significant and resulting in a full mediation. As the spread of COVID-19 continues, depression from COVID-19 affects people’s mind-set, which may create long-term problems in the minds of future workforces.

## Limitations

This study was conducted in a situation, where almost all the countries of the universe were literally locked down. So, it is not possible to get a larger sample size. Furthermore, most of the respondents were residing in the urban or semi-urban areas while participating in the survey. So, the research result may not be generalized in all over the world.

## Conclusion and implication

This research results empirically found that due to the outbreak of COVID-19 human emotion significantly stuck with different negative aspects. Thus, the fear turns into depression and the future workforce is going through an unimaginable tough situation. Mental health and physical health are also seriously affected by distress and long-term negativity (Mattioli & Ballerini Puviani, 2020). As the world’s economy had already stumbled and no one actually knows how the world will be retrieved from this unwanted situation, the economy will be squeezed in many folds all over the world. Therefore, the policymakers should consider these research findings while making an economic revival plan and offer some forms of special incentive packages to flourish the potentiality of the relatively younger working class to boost up the world’s economy.

## Appendix

**Appendix 1.** Measurement items.

Fear of COVID-19 (Ahorsu et al., 2020)	FCV19 S1	I am most afraid of the novel coronavirus.
	FCV19 S2	It makes me uncomfortable to think about novel coronavirus.
	FCV19 S3	My hands become sweaty when I think about COVID-19.
	FCV19 S4	I am afraid of losing my life because of COVID-19.
	FCV19 S5	When watching news and stories about novel coronavirus on social media or any other media (i.e. TV, Radio), I become nervous or anxious.
	FCV19 S6	I cannot sleep because I am worried about getting the novel coronavirus.
	FCV19 S7	My heart races or palpitates when I think about getting COVID-19.
Depression from COVID-19 (Le et al., 2017; Shea et al., 2009)	DEP1	I could not seem to experience any positive feelings at all because of the fear of COVID-19.
	DEP2	I find it difficult to work up the initiative to do things due to the fear of COVID-19.
	DEP3	I felt down-hearted and blue due to the fear of COVID-19.
	DEP4	I was unable to become enthusiastic about anything in this pandemic situation.
	DEP5	I felt I was not worth much as a person in this pandemic situation.
	DEP6	I felt that life is meaningless due to the fear of COVID-19.
Future Career Anxiety (Tsai et al., 2017)	FCA1	I worry about future employment because of a potential economic recession due to the outbreak of COVID-19.
	FCA2	I worry about future employment because of fierce competition in the job market due to the outbreak of COVID-19.
	FCA3	I worry about future employment because my salary would probably not be as excellent as I wish for the devastating effect of COVID-19.
	FCA4	I worry about future employment because of the increasing unemployment and job cut reported by the mass media for the reason of COVID-19.
	FCA5	I worry about future employment because I probably would not find a job that interests me for the reason of COVID-19.

DEP: depression from COVID-19; FCA: future career anxiety; FCV19S: fear of COVID-19 scale.
